# Arteriovenous malformation of the external ear: a clinical assessment with a scoping review of the literature^☆^

**DOI:** 10.1016/j.bjorl.2016.09.004

**Published:** 2016-10-17

**Authors:** Shin Hye Kim, Seung Hoon Han, Yoonjae Song, Chang Sik Park, Jae-Jin Song

**Affiliations:** a Korea University Medical Center, Korea University College of Medicine, Department of Otorhinolaryngology-Head and Neck Surgery, Seoul, Republic of Korea; b Seoul National University Bundang Hospital, Seoul National University College of Medicine, Department of Otorhinolaryngology-Head and Neck Surgery, Seongnam, Republic of Korea; c Seoul National University Bundang Hospital, Seoul National University College of Medicine, Department of Plastic Surgery, Seongnam, Republic of Korea

**Keywords:** Ear, Tinnitus, Arteriovenous malformations, Embolization, therapeutic, Surgical procedures, operative, Orelha, Zumbido, Malformações arteriovenosas, Embolização, terapêutica, Procedimentos cirúrgicos, operatórios

## Abstract

**Introduction:**

Auricular Arteriovenous Malformation of the external ear is a rarely encountered disease; in particular, arteriovenous malformation arising from the auricle, with spontaneous bleeding, has seldom been reported.

**Objective:**

In the current study, we report an unusual case of late-onset auricular arteriovenous malformation originating from the posterior auricular artery that was confirmed by computed tomographic angiography. The case was successfully managed by pre-surgical intravascular embolization followed by total lesion excision. Prompted by this case, we also present a scoping review of the literature.

**Methods:**

A case of a 60 year-old man with right auricular arteriovenous malformation treated in our tertiary care center, and 52 patients with auricular arteriovenous malformation described in 10 case reports and a retrospective review are presented. Auricular arteriovenous malformation can manifest as swelling of the ear, pulsatile tinnitus, pain, and/or bleeding. On physical examination, a pulsatile swelling and/or a tender mass is evident. When arteriovenous malformation is suspected, the lesions should be visualized using imaging modalities that optimally detect vascular lesions, and managed via embolization, mass excision, or auricular resection. Effectiveness of the various diagnostic methods used and the treatment outcomes were analyzed.

**Results:**

Various imaging modalities including Doppler sonography, computed tomographic angiography, magnetic resonance angiography, and/or transfemoral cerebral angiography were used to diagnose 38 cases reported in the literature. In another 15 cases, no imaging was performed; treatment was determined solely by physical examination and auscultation. Of the total of 53 cases, 12 were not treated (their symptoms were merely observed) whereas 20 underwent therapeutic embolization. In total, 32 patients, including 1 patient who was not treated and 10 with persistent or aggravated arteriovenous malformation after previous embolization, underwent mass excision or auricular resection depending on the extent of the lesion. No major postoperative complication was recorded. The postoperative follow-up duration varied from 1 month to 19 years, and only one case of unresectable, residual cervicofacial arteriovenous malformation was recorded.

**Conclusion:**

Auricular arteriovenous malformation is a rarely encountered disease, but should be suspected if a patient presents with a swollen ear and pulsatile tinnitus. Appropriate imaging is essential for diagnosis and evaluation of the extent of disease. As embolization affords only relatively poor control, total surgical removal of the vascular mass is recommended.

## Resumo

**Introdução:**

Malformação Arteriovenosa Auricular da orelha externa é uma doença raramente observada, em particular, na região da aurícula, com hemorragia espontânea, tem sido infrequentemente relatada.

**Objetivo:**

No presente estudo, relatamos um caso incomum de malformação arteriovenosa auricular de início tardio originária da artéria auricular posterior confirmada por angiotomografia computadorizada. O caso foi controlado com sucesso por embolização endovascular pré-cirúrgica seguida por excisão completa da lesão. Além disso, nós também apresentamos uma revisão abrangente da literatura.

**Método:**

Um homem de 60 anos de idade com malformação arteriovenosa auricular direita tratado em nosso centro de atendimento terciário e 52 pacientes com malformação arteriovenosa auricular descritos em 10 relatos de casos e um estudo de revisão são apresentados. A malformação arteriovenosa auricular pode manifestar-se como inchaço da orelha, zumbido pulsátil, dor e/ou sangramento. Ao exame físico, um edema pulsátil e/ou uma massa sensível é evidente. Quando há suspeita de malformação arteriovenosa, as lesões devem ser visualizadas usando modalidades de imagem que detectam de maneira ideal as lesões vasculares, e tratadas por meio de embolização, excisão total da lesão, ou ressecção auricular. A eficácia dos vários métodos de diagnóstico utilizados e os desfechos do tratamento foram analisados.

**Resultados:**

Várias modalidades de imagem, incluindo ultrassonografia Doppler, angiotomografia computadorizada, angiografia por ressonância magnética e/ou angiografia cerebral transfemoral foram usadas para diagnosticar 38 casos relatados na literatura. Em outros 15 casos, nenhuma imagem foi realizada; o tratamento foi determinado unicamente pelo exame físico e ausculta. Do total de 53 casos, 12 não foram tratados (os seus sintomas foram apenas observados), enquanto que 20 foram submetidos a embolização terapêutica. No total, 32 doentes, incluindo um paciente que não foi tratado e 10 com malformação arteriovenosa persistente ou agravada após a embolização anterior, foram submetidos a excisão completa da lesão ou ressecção auricular, dependendo da extensão da lesão. Nenhuma complicação pós-operatória importante foi registrada. O tempo de seguimento pós-operatório variou de 1 mês a 19 anos, e apenas um caso de malformação arteriovenosa cervicofacial irressecável, residual foi registrado.

**Conclusão:**

A malformação arteriovenosa auricular é uma doença raramente encontrada, mas deve ser suspeitada se um paciente apresentar orelha inchada e zumbido pulsátil. A imagem apropriada é essencial para o diagnóstico e avaliação da extensão da doença. Como a embolização proporciona apenas um controle relativamente precário, a remoção cirúrgica total da lesão vascular é recomendada.

## Introduction

An Arteriovenous Malformation (AVM) is an abnormal connection between one or more arteries and veins, bypassing the capillary system.^1^ In most cases, the AVM arises from the intracranial area, but occasionally originates from extracranial vessels.^2^ In a retrospective review of 81 patients with AVM of the head-and-neck, the most common site was the cheek (31%), followed by an ear (16%).^3^ The AVM is almost always present at birth, but manifests later in life.

Here, we report an unusual case of late-onset auricular AVM originating from the posterior auricular artery. Also, a scoping review of the literature allows us to discuss the roles played by imaging in the diagnosis and management of AVM.

## Materials and methods

### Search criteria

We adhered to the PRISMA (Preferred Reporting Items for Systematic Reviews and Meta-Analyses) guideline when performing PubMed (http://www.ncbi.nlm.nih.gov/pubmed/) searches to identify all studies on AVM of the external ear.^4^ The keywords used were “arteriovenous malformation” and “ear”, and the search was limited to articles in the English language. AVMs originating from the external auditory canal, or the pre- or retro-auricular areas, were excluded.

### Our case and literature review

We treated a 60 year-old man with right-side auricular AVM. The study was approved by the institutional review board of the Clinical Research Institute at our center (B-1601-329-002). The literature review identified a total of 52 AVM patients described in 11 reports (10 case reports and 1 retrospective review). The 10 case reports dealt with 11 cases of auricular AVM and the single review analyzed 41 cases. The chief complaints, duration of symptoms, events that aggravated the AVMs, and history of spontaneous AVM bleeding were analyzed.

### Diagnostic process and differential diagnosis

The laterality, location, and extent of AVM were investigated. Various diagnostic imaging modalities including Doppler Sonography (DS), Temporal Bone Computed Tomographic Angiography (TBCTA), Magnetic Resonance Angiography (MRA), and/or Transfemoral Cerebral Angiography (TFCA) were employed in individual cases, and the findings of the key diagnostic modalities were reviewed. In patients who underwent diagnostic angiography, the main arteries and feeder vessels are summarized.

### Management options and treatment outcomes

The AVM management options included observation, embolization, mass excision, or auricular resection. After the treatment, the clinical condition was described as controlled, improved, persistent, or aggravated. We also explored the application of reconstructive procedures such as Skin-Thickness Split Grafting (STSG) and total auricular reconstruction. Postoperative complications, follow-up durations, and final status were reviewed.

## Results

### Our experience with late-onset AVM

A 60 year-old man visited the emergency department with massive spontaneous bleeding from the right ear. He had a history of recurrent swelling of the right auricle, and pulsatile tinnitus. Three years prior, the patient had visited our outpatient clinic with recurrent auricular swelling, and was diagnosed with an otohematoma because of the cauliflower-like appearance of the auricle.

**Figure 1:**
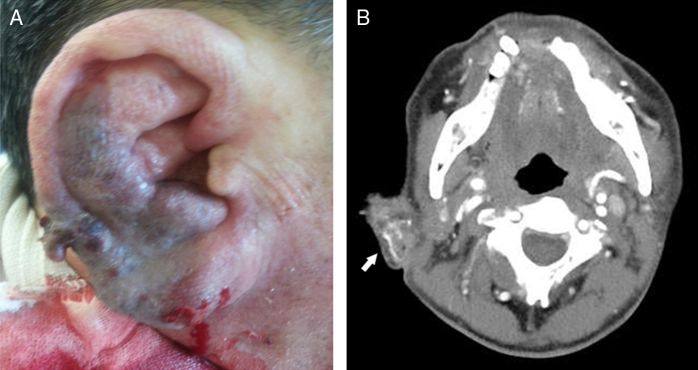
Gross findings and temporal bone computed tomographic angiography findings on the ear of a 60 year-old man, as recorded in the emergency room: (A) the patient presented with a swollen ear and spontaneous massive bleeding; (B) temporal bone computed tomographic angiography revealed a right-side, auricular vascular tangled lesion (white arrow).

**Figure 2:**
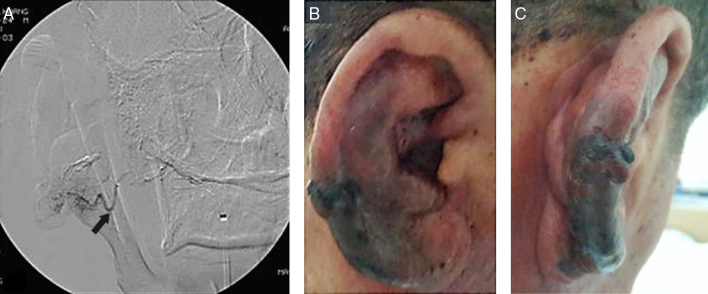
Preoperative therapeutic embolization using transfemoral cerebral angiography and gross ear findings 3 days after embolization: (A) transfemoral cerebral angiography revealed large tortuous vessels and innumerable small vessels. The principal feeder vessel of the arteriovenous malformation originated from the posterior auricular artery (black arrow), and was completely occluded with glue; (B) and (C) after embolization, the bleeding stopped, but ischemic necrosis of the skin progressed.

**Figure 3:**
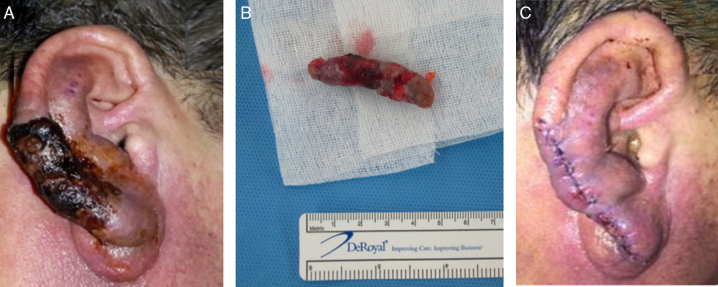
Gross ear findings at 2 weeks after embolization and total excision of the arteriovenous malformation. (A) Two weeks after transarterial embolization, the boundary of the necrotic skin lesion became distinct. (B) The necrotic skin and the mass of the arteriovenous malformation was excised under local anesthesia. The dimensions of the mass were 4.8 cm × 1.2 cm × 1.2 cm. (C) The auricle was closed under minimal tension.

On physical examination, the right auricle exhibited excessive swelling and discoloration (Fig. 1A). The tympanic membrane and external auditory canal were normal. TBCTA revealed enlargement of the right helix, and an entangled vascular lesion (Fig. 1B). We suspected an auricular AVM and performed transarterial embolization using TFCA. The principal AVM-feeding artery originated from the posterior auricular artery, and we completely embolized the artery with glue (Fig. 2A). After embolization, bleeding was controlled, but ischemic necrosis of the auricular skin developed (Fig. 2B and C). Two weeks later, the necrotic skin boundary became distinct (Fig. 3A), and we thus planned total mass excision.

Under local anesthesia, the skin was incised along the vertical plane of the right auricle and the AVM mass was totally excised (Fig. 3B). The adjacent necrotic skin was excised and primary closure was performed (Fig. 3C). The patient recovered without any complications. At 6 months postoperatively, the auricle was well-healed without any evidence of recurrence.

### Demographics and symptoms of AVM patients

Table 1 summarizes the findings of 12 studies on 53 cases of auricular AVM.^1^, ^5^, ^6^ Of 11 case reports, 7 were men and 4 were women, with a median age of 21 (15–45) years. In the retrospective review of 41 cases, the male-to-female patient ratio was not mentioned, and the mean age was 26 (1–55) years. Chief complaints were swelling of the ear, pulsatile tinnitus, intermittent pain, hearing loss, and/or profuse spontaneous bleeding. About two-thirds of patients reported that their symptoms had commenced at a very young age. Seven patients reported that their AVMs became aggravated during puberty, and 6 had histories of trauma.

**Table 1:** Summary of cases showing arteriovenous malformation of the external ear from previous reported literatures and this study.

	Ramadass T (2000)^9^	Pham TH (2001)^12^	Wu JK (2005)^5^	Meher R (2008)^11^	Saxena SK (2008)^10^
No of patients	1	1	41	2	1
Age	25Y	41Y	26Y (1Y–55Y)	16Y, 22Y	21Y
Sex	M	M	Not described	M, F	F
Nations	Bangladesh	USA	USA and France	India	India
Chief complaint	Ear swelling, profuse bleeding	Ear swelling, PT intermittent bleeding	PT (51.2%), bleeding (41.5%), pain (29.3%), bruit/thrill (24.4%)	Pt.1: Intermittent pain/Pt.2: ear swelling, PT	Ear swelling, PT
Duration of symptom	7Y	6Y		10Y, 15Y	
Bleeding history	Several times	Several times		Pt.2: 2 times	None
Lesion site	R) auricle	L) auricle	Auricle and extraauricular involvement (Retroauricular: 46.3%, Neck: 22%, None: 22%)	L) auricle, R) auricle	R) auricle
Key diagnostic study/findings	A/enlarged and tortuous vessels	A/diffuse network of shunts	A and/or MRI: 65.9%	Pt.1: DS/multiple dilated anechoic areas, Pt.2: MRA/enlarged serpiginous structures	A/diffuse shunts with STA
Main feeding vessels	PAA, OA	PAA, STA, OA	PAA, STA, OA	Pt.2: PAA, STA	STA
Treatment	Excision and STSG, ear elevation after 4M	Excision, and STSG after embolization	Observation (*n* = 12, 29.3%), Embolization (*n* = 9, 21.9%), Auricular resection c/w embolization (*n* = 20, 48.8%)	Pt.1,2: Excision and STSG	Failed embolization, excision after feeding artery ligation
Follow-up duration/Final status	4M/follow-up lost after 2^nd^ operation	2Y/no recurrence	5Y (1–19Y)/20 patients with amputation: controlled (*n* = 16), improved (*n* = 3), persistent (*n* = 1)		3Y/no recurrence

	Woo HJ (2008)^13^	Whitty LA (2009)^8^	Prasad KC (2011)^14^	Goel A (2011)^15^	Meena BK (2013)^1^	Dixit SG (2013)^6^	Kim SH (this study)
No of patients	1	1	1	1	1	1	1
Age	20Y	15Y	45Y	22Y	21Y	21Y	60YA
Sex	M	M	M	F	F	M	M
Nations	Korea	USA	India	India	India	India	Korea
Chief complaint	PT	Ear swelling, intermittent pain	Ear swelling	Ear swelling, PT	Ear swelling, PT	Ear swelling	Ear swelling, massive bleeding, PT
Duration of symptom	7M	2Y	2-3Y	4Y	1Y	Since birth	3Y
Bleeding history		None		2 times	2 times	None	
Lesion site	L) auricle	L) auricle		R) auricle	L) auricle		R) auricle
Key diagnostic study/findings	MRI/abnormal signal voiding intensity of the mass	Auscultation/audible bruit	A/AVM mass and PAA aneurysm	DS	DS,CTA/enlarged serpiginous structures	MRA/abnormal tortuous vessel	CTA/inner vascular tangled lesion
Main feeding vessels	PAA, STA		PAA		PAA, STA	PAA	PAA
Treatment	Failed embolization, excision	Excision	Vessel ligation and mass excision	Excision and STSG	Excision	Embolization under external approach	Excision after embolization
Follow-up duration/final status	2Y/no recurrence	1M/no recurrence	2Y/no recurrence				3M/no recurrence

Y, year; M, month; R, right; L, left; PT, pulsatile tinnitus; A, angiography; CTA, computed tomography angiography; MRA, magnetic resonance angiography; DS, Doppler sonography; PAA, posterior auricular artery; STA, superficial temporal artery; OA, occipital artery; AVM, arteriovenous malformation; STSG, Split-Thickness Skin Graft.

### Diagnosis

Table 1 shows key diagnostic study and their findings. In 38 of the 53 cases, DS, TBCTA, MRA, and/or TFCA were silent in terms of diagnosis. No imaging was performed in the other 15 cases; treatment was determined solely by physical examination and auscultation.^5^, ^7^ On angiography, the most common feeding vessels were the posterior auricular, superficial temporal, and occipital arteries.

### Management and treatment outcomes

**Figure 4:**
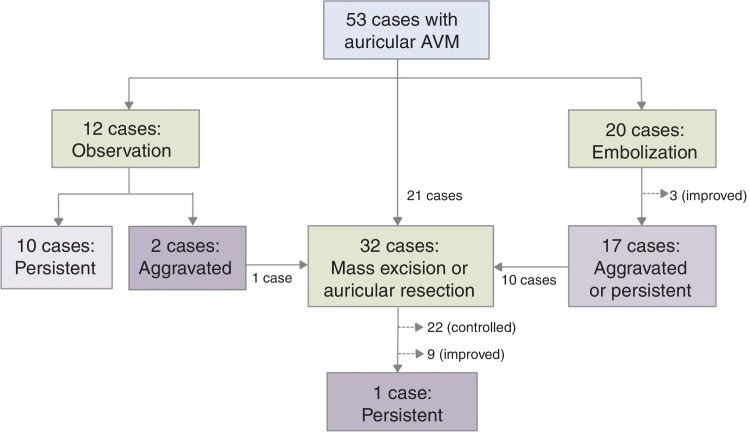
Flow chart of the treatment of 53 patients with auricular arteriovenous malformations.

The flow chart of Fig. 4 shows the treatment options and outcomes. Twelve of 53 patients were initially followed-up without any treatment. Of these, AVMs persisted in 10, and became aggravated in 2. Of the latter 2, one underwent auricular resection but the other rejected an operation. Twenty patients underwent therapeutic embolization. Of these, the AVMs improved in 3, but became aggravated in 17. Of the latter 17, 10 underwent AVM mass excision or auricular resection.

A total of 21 patients underwent AVM mass excision or auricular resection as initial treatment. Of these patients, plus a further 11 who underwent excision or resection as salvage management, 22 (69%) were controlled and 9 (28%) improved, and only one had a persistent unresectable AVM in the adjacent region. In these 32 patients, wound closure was performed by simple linear closure (*n* = 18); STSG (*n* = 10); immediate reconstruction using salvaged auricular cartilaginous framework covered by temporoparietal flap (*n* = 1), expanded local flap (*n* = 1), advancement flap (*n* = 1), and free flap (*n* = 1). One patient underwent a two-staged operation: auricular resection and STSG initially and ear lobule creation and ear elevation secondarily.^8^ No major postoperative complication was noted, except for transient tinnitus in one patient and skin necrosis at the site of wound closure in two.^7^ The postoperative follow-up duration varied from 1 month to 19 years, and only one case had residual cervicofacial AVM.^7^

## Discussion

Schobinger classified AVM into four stages. The symptoms of stage I (quiescence) are warm and discolored skin; those of stage II (expansion) are bruit, pulsation, and swelling. Stage III (destruction) is characterized by pain, ulceration, and bleeding; whereas stage IV (decompensation) features cardiac failure.^3^, ^9^ The presenting signs and symptoms correlate with the stage of AVM. Although many AVMs are asymptomatic, they may alternatively trigger severe pain and/or bleeding. The most common symptoms are pulsation (51.2%), bleeding (41.5%), and pain (29.3%).^7^ Hearing can also deteriorate, presumably because the bruit is audible.^10^, ^11^, ^12^, ^13^ There are 2 types of AVM with regard to the flow rate: fast-flowing and slow-flowing.^14^ Most of fast-flowing regions are arteriovenous fistulas whereas slow-flow AVMs are produced by venous, capillary, or lymphatic lesions.^14^ This flow rate-based classification may be of importance as different treatment options are needed for the 2 types.

Enlargement of an AVM may be triggered by trauma, infection, or hormonal influences.^15^ In the review paper of 41 AVM patients, expansion occurred during childhood in 7, adolescence in 14, pregnancy in 10, and adulthood in 10.^7^ Some AVMs may remain quiescent until adolescence and, even into adulthood. Our patient was rather older than those of other reports. Moreover, we had earlier misdiagnosed the lesion as an otohematoma. AVM is similar to cauliflower ear in that both diseases may present with a swollen deformed auricle. As simple drainage based on a misdiagnosis of otohematoma may trigger massive bleeding in a case of AVM, differential diagnosis of AVM from an otohematoma is important.

In two case reports, surgical intervention was planned under suspicion of AVM in the absence of imaging data.^2^ However, imaging modalities are required for accurate diagnosis of the vascular lesion and confirmation of the extent. MRA or TFCA revealed total auricular involvement in 24 (89%), although 56.1% of 27 patients were thought to exhibit only partial involvement upon physical examination.^7^ Although conventional TFCA is invasive, the technique is useful to identify the principal feeding vessel and an appropriate embolization portal.^15^ TFCA-guided embolization is a useful initial therapeutic step, but surgical excision is required in most cases to avoid recurrence.

Treatment is unnecessary (especially in children) if the AVM is small and asymptomatic. In an untreated group of 12 patients, 2 of stage I and 7 of stage II remained stable.^3^, ^7^ If the AVM is symptomatic, complete excision (preceded by embolization) is the treatment of choice.^6^ Ligation of the arterial vessels alone or partial excision should be avoided, because a new collateral circulation will form, triggering further enlargement.^15^, ^16^ In our scoping review, 17 of 20 patients who underwent initial embolization exhibited aggravated or persistent AVMs. Wu et al. reported that the mean lag time between the last embolization and ultimate resection was 5.6 years (range: 2–8 years).^5^ Based on these results, embolization is best employed only to reduce blood loss and facilitate surgical extirpation.

## Conclusion

Our case was initially misdiagnosed as an otohematoma because of the cauliflower-like auricle, and the patient presented 3 years later with massive bleeding. To accurately diagnose an AVM, meticulous physical examination including palpation and auscultation and imaging are essential. In most cases of auricular AVM, optimal treatment is a combination of super-selective embolization and complete surgical excision.

## Funding

This work was supported by a grant from the Korea Health Technology R&D Project through the Korea Health Industry Development Institute (KHIDI), funded by the 10.13039/501100004726Ministry of Health & Welfare, Republic of Korea (grant number HI14C2264).

## Conflicts of interest

The authors declare no conflicts of interest.
